# Learning from Co-expression Networks: Possibilities and Challenges

**DOI:** 10.3389/fpls.2016.00444

**Published:** 2016-04-08

**Authors:** Elise A. R. Serin, Harm Nijveen, Henk W. M. Hilhorst, Wilco Ligterink

**Affiliations:** ^1^Wageningen Seed Lab, Laboratory of Plant Physiology, Wageningen UniversityWageningen, Netherlands; ^2^Laboratory of Bioinformatics, Wageningen UniversityWageningen, Netherlands

**Keywords:** co-expression, gene expression, gene networks, gene prioritization, transcriptomics

## Abstract

Plants are fascinating and complex organisms. A comprehensive understanding of the organization, function and evolution of plant genes is essential to disentangle important biological processes and to advance crop engineering and breeding strategies. The ultimate aim in deciphering complex biological processes is the discovery of causal genes and regulatory mechanisms controlling these processes. The recent surge of omics data has opened the door to a system-wide understanding of the flow of biological information underlying complex traits. However, dealing with the corresponding large data sets represents a challenging endeavor that calls for the development of powerful bioinformatics methods. A popular approach is the construction and analysis of gene networks. Such networks are often used for genome-wide representation of the complex functional organization of biological systems. Network based on similarity in gene expression are called (gene) co-expression networks. One of the major application of gene co-expression networks is the functional annotation of unknown genes. Constructing co-expression networks is generally straightforward. In contrast, the resulting network of connected genes can become very complex, which limits its biological interpretation. Several strategies can be employed to enhance the interpretation of the networks. A strategy in coherence with the biological question addressed needs to be established to infer reliable networks. Additional benefits can be gained from network-based strategies using prior knowledge and data integration to further enhance the elucidation of gene regulatory relationships. As a result, biological networks provide many more applications beyond the simple visualization of co-expressed genes. In this study we review the different approaches for co-expression network inference in plants. We analyse integrative genomics strategies used in recent studies that successfully identified candidate genes taking advantage of gene co-expression networks. Additionally, we discuss promising bioinformatics approaches that predict networks for specific purposes.

## Introduction

In plants, the age of systems biology has accelerated the investigation of complex molecular mechanisms underlying intricate developmental and physiological processes. Since plants are anchored to their environment, they cannot escape from stresses by simply moving away. Instead, plants have developed a wide range of mechanisms to cope with environmental fluctuations. This plasticity generally involves changes at the level of DNA, RNA, protein and metabolites, resulting in complex phenotypes governed by multiple genes. Advanced genetic and molecular tools have led to tremendous progress in revealing the genetic architecture but also the regulatory mechanisms of complex traits (Mochida and Shinozaki, 2011). The development of molecular profiling techniques nowadays enables the high-throughput and affordable acquisition of large omics data sets, such as for transcriptomics, proteomics and metabolomics.

While substantial efforts are being made to generate large omics data sets, there is a growing need to develop platforms to integrate these data and derive models describing biological interactions in plants. In this context, networks have rapidly become an attractive approach to manage, display and contextualize these large data sets in order to obtain a system level and molecular understanding of biological key processes (Barabási and Oltvai, 2004; Usadel et al., 2009; Costa et al., 2015; Silva et al., 2016).

Biological networks are generally classified by the nature of the compounds and interactions involved. These networks can be derived from various molecular data resulting in, e.g., gene expression networks (correlation or co-expression networks), protein-protein interaction (PPI) networks, metabolic networks and signaling networks. Graphically, networks are represented as an ensemble of components (nodes or vertices) and interactions depicted by links (edges) connecting pairs of nodes. Such interaction maps provide an attractive framework to study the organizational structure of complex systems and have found many applications in plants (Jiménez-Gómez, 2014).

**Figure 1:**
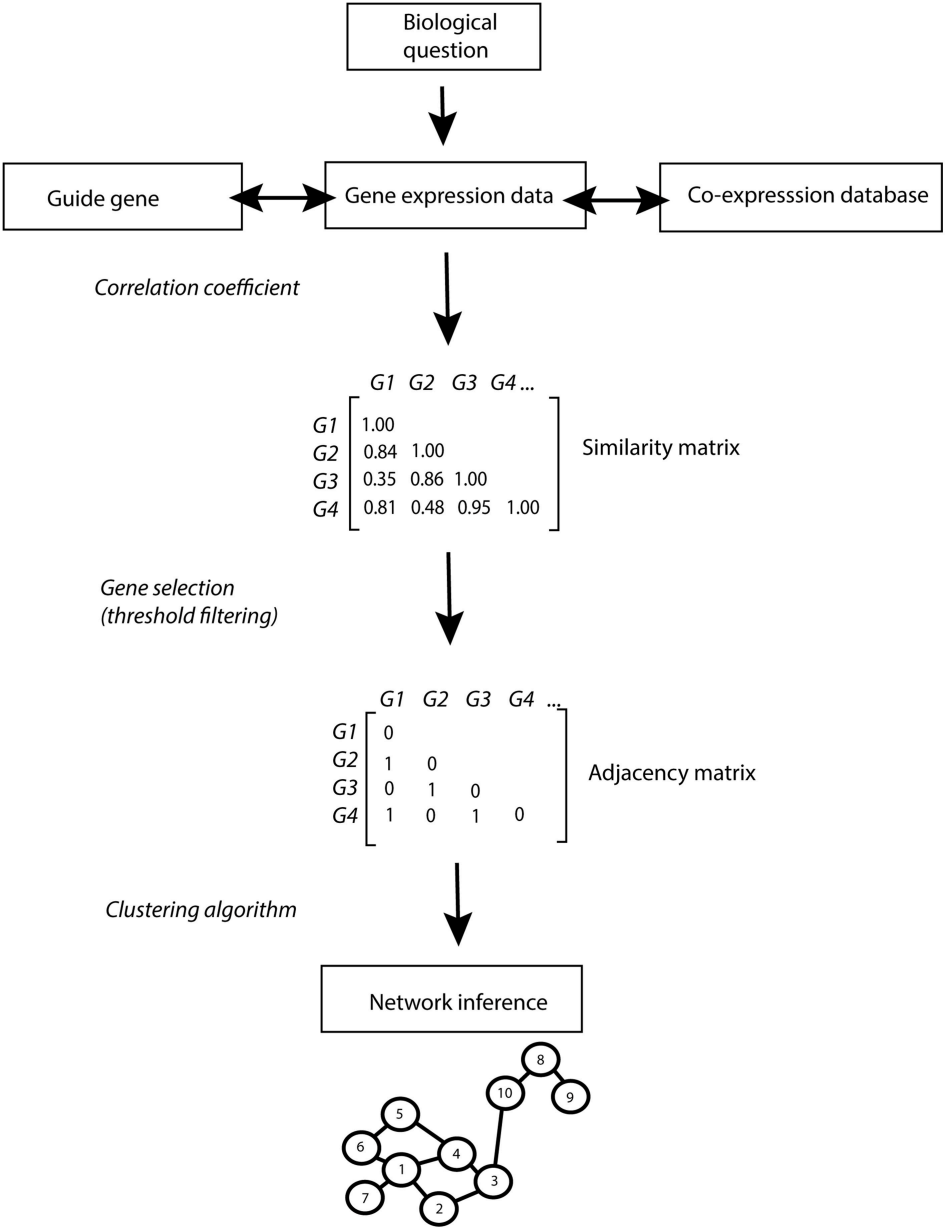
**Co-expression network inference pipeline**. The biological question addressed drives the strategy for the co-expression network analysis: prior knowledge can be used to identify guide-genes and co-expression databases can be queried to investigate gene co-expression patterns across multiple conditions. Similarity in gene expression patterns is calculated using correlation coefficients (Pearson, Spearman…). A user defined threshold (in this example set at 0.8) enables the selection of genes with high co-expression scores. Significantly co-expressed genes are reported in the binary adjacency matrix as 1. A clustering algorithm is applied on the adjacency matrix to infer networks of significantly co-expressed genes. In the resulting network, significantly co-expressed genes are depicted as numbered nodes (vertices) linked by edges (links). The length of the edges is relative to the expression similarity of the connected genes, with a short edge corresponding to a high co-expression value. A “path” corresponds to the number of edges connecting two nodes (the shortest path from node 9 to 4 is 4 edges). Hubs are identified as highly connected nodes (node 1) and group of connected genes form modules (nodes 1–7). Network properties can be described by different parameters such as: •The **connectivity** of a network corresponds to the total number of links in the network. •The **node degree** corresponds to the number of connections of a node with other nodes in the network (node 4 has a node degree of 3). •The **betweenness** of a node corresponds to the sum of the shortest paths connecting all pair of nodes in the network, passing through that specific node. The betweenness of node 8 corresponds to the sum of the shortest path the connecting node 10–9, 3–9, 4–9 etc…).

The fast development of transcriptomic technologies, as compared to other analytical platforms, has supported a range of studies on genetic and environmental perturbations at the transcriptome level in many organisms. Co-expression networks have grown in popularity in the last years as they enable the integration of large transcriptional data sets (Li et al., 2015; Liseron-Monfils and Ware, 2015). Co-expression network analysis allows the simultaneous identification, clustering and exploration of thousands of genes with similar expression patterns across multiple conditions (co-expressed genes). The main procedure for co-expression network inference is explained in Box 1 and illustrated in Figure 1. Briefly, a similarity score (i.e., correlation coefficient) is calculated from the pairwise comparison of the gene expression patterns for each possible pair of genes. Above a certain threshold, genes and gene pairs form a list of nodes and corresponding edges from which the network is constructed. As a rule, the guilt-by-association principle is applied stating that genes sharing the same function or that are involved in the same regulatory pathway will tend to present similar expression profiles and hence form clusters or modules in the network (Wolfe et al., 2005). Thus, within the same module, genes of known function can be used to predict the function of co-expressed unknown genes (Rhee and Mutwil, 2014).

Box 1 **Network Inference**

Constructing a network of genes from expression data generally consists of the following steps: first a measure of similarity or relatedness is calculated for each of the possible gene pairs. The resulting list of gene pairs is then filtered using a threshold value for the similarity score. The remaining gene pairs form a list of edges from which the network is constructed (Figure 1). As an optional next step, modules of highly related genes can be extracted from the network using gene prioritization approaches.

**Similarity Score**

Gene expression values are usually log_2_ transformed before calculating the similarity score in order to scale the values to the same dynamic range.

Several measures are used to determine a similarity score between gene pairs, each with its specific strengths and weaknesses. Simple Pearson or Spearman correlation is often used and performs well compared to more sophisticated methods, both in terms of finding gene relationships and performance on large data sets (Song et al., 2012; Ballouz et al., 2015). Pearson is the most popular correlation measure, although it assumes a linear correlation, normally distributed values and is sensitive to outliers. Spearman's rank correlation is more robust, but also less powerful. Another often used measure that can describe non-linear relations between genes is called Mutual Information (MI) (Meyer et al., 2008). Song et al. (2012) found that in many situations MI does not perform better than correlation. They proposed “bi-weight mid-correlation” (bicor) as an attractive alternative correlation measure that is more robust than Pearson correlation.

**Significance Threshold**

When the similarity scores between all gene pairs have been determined, a cutoff is applied to select the gene pairs that should be connected in the network. This can be an arbitrary cutoff, but there are several ways to make a more informed choice. Lee et al. (2004) selected only the top 0.5% most positively and the top 0.5% most negatively correlated pairs. Bassel et al. (2011) chose a cutoff that results in a network following a power-law distribution, using the Weighted Gene Co-expression Network Analysis (WGCNA) package (Langfelder Langfelder and Horvath, 2008). Butte and Kohane (2000) used random permutations of the expression data to determine a cutoff for significant interactions. Other approaches calculate a *p*-value based on the null hypothesis that the correlation between two genes is 0.

Zhang and Horvath (2005) proposed to use soft thresholds instead of hard cutoffs, to produce weighted gene networks and preserve the underlying continuous nature of the correlation. However, visualizing these networks is challenging since the directly linked neighbors of a node are difficult to identify.

**Promising Approaches**

Correlation networks do not distinguish between direct and indirect interactions. The ARACNE algorithm (Margolin et al., 2006; Meyer et al., 2008) addresses this by pruning edges based on the analysis of gene triplets. If genes A, B, and C are fully connected in the network and the edge between A and C has the lowest weight, this edge could actually be an indirect interaction of A and C through B.

Correlation networks have undirected edges, since no causality can be inferred from two connected genes, although work has been published to address this (Opgen-Rhein and Strimmer, 2007). Regression methods are well-suited to find directed edges, since they try to find the set of genes that best predict the expression of a given target gene. However, because regression methods are generally computational demanding, the set of possible predictor genes is often limited to known transcription factors (Vignes et al., 2011; Marbach et al., 2012). In addition, Bayesian networks also allow the inclusion of prior knowledge, but their application is even more computationally challenging and not feasible for large sets of genes (Tamada et al., 2003; Imoto et al., 2004; Werhli and Husmeier, 2008).

The two main applications for co-expression network analysis are to find novel genes involved in the biological process under investigation and to suggest the biological process a gene is involved in. Intuitively, reliable networks are needed to infer meaningful gene function predictions. Such networks heavily depend on a combination of decisions taken throughout the network inference process. From the quality, type and availability of the input data, the correlation coefficient and inference algorithm used, to the prior knowledge, the experimental and computational resources, any negligence can result in unreliable networks and subsequent misleading biological interpretations.

Caveats and opportunities of co-expression network analyses have been discussed previously (Usadel et al., 2009). When handling large data sets, co-expression networks can become very complex which limits their biological interpretation (Usadel et al., 2009). In addition, in contrast to regulatory networks, and because of their static representation, co-expression networks do not provide *per se* information on the nature of the regulatory relationship of connected genes (Stuart et al., 2003). Careful application of network analysis tools and strategies is thus important to maximize the information extraction, to disentangle reliable network connections and to infer true biological meaning.

In this review, we aim to provide an overview of the different strategies to employ during or after the co-expression network construction with the common aim of exploiting the full predictive potential of co-expression networks. The application of these strategies is illustrated by examples of recent studies. Particular attention is given to available and promising bioinformatics tools. Finally, we will speculate on network aspects worth developing in the near future to strengthen their inference power for a comprehensive understanding of the regulation of important biological processes.

## Data availability for co-expression network analysis

In the post-genomic era, the reduction of costs for large scale and high-throughput measurement technologies, such as for transcriptomics, has to the extensive collection of gene expression profiles capturing changes in gene expression during development, between different treatments or tissues, etc.

In addition, the sequenced genomes of model plants (e.g., Arabidopsis, medicago, and poplar) and economically important crops (e.g., tomato, potato, tobacco, rice, and soybean) strongly improve our understanding of transcriptional dynamics.

The compendia of generated data led to the development of publicly available gene expression databases (Table 1). These databases still largely contain microarray data and many of them are related to the model plant Arabidopsis. In recent years, RNA-sequencing, using next-generation high-throughput sequencing technologies (RNA-seq) has proven to be a powerful tool for whole transcriptome profiling with enhanced sensitivity for the discovery of new transcripts and enhanced specificity such as for the examination of allele-specific expression. The power of these sequencing technologies has enabled co-expression network analysis in species without a sequenced genome and, as a result, has opened the way for new applications (see Section Comparative Co-expression Network Analysis). RNA-seq based co-expression network construction is still in its infancy (Iancu et al., 2012; Ballouz et al., 2015) but the foreseen predominance of next generation sequencing tools in the coming years will certainly enrich existing databases for the benefit of network studies. Microarrays are still commonly used for transcriptome analysis because they are relatively cheap and their analysis is highly standardized. Comprehensive microarray gene expression sets are available in public repositories such as the Gene Expression Omnibus (GEO, Edgar et al., 2001), Genevestigator (Hruz et al., 2008) or Array Express (Parkinson, 2004). Other tools, such as the online bio-analytical resource for plant biology (BAR, Winter et al., 2007), provide interactive interfaces for the exploratory visualization of gene expression variation.

**Table 1:** **Overview of available resources for co-expression network analysis**.

	**Resources**	**Description**	**Target species**	**Link**	**References**
Data availability and data selection for co-expression network analysis	**Search Engine for Gene Expression**				
	BAR—eFP browser	Interactive visualization of gene expression	Arabidopsis	http://bar.utoronto.ca/	Winter et al., 2007
	GEO	Public functional genomics data repository	Several species	http://www.ncbi.nlm.nih.gov/geo/	Edgar et al., 2001
	Genevestigator	Database for curated gene expression data	Several species	http://www.plexdb.org/plex.php?database=Arabidopsis	Hruz et al., 2008
	Phytozome	Comparative platform for plant genomics	Several species	http://phytozome.jgi.doe.gov/pz/portal.html	Goodstein et al., 2012
	ArrayExpress	Database for large functional genomics	Several species	http://www.ebi.ac.uk/arrayexpress/	Brazma, 2003
	**Web-Interfaces for Co-Expression Analysis**				
	ATTED-II	Gene co-expression database	Several species	http://atted.jp/	Obayashi et al., 2007, 2014
	Cressexpress	Co-expression analysis for Arabidopsis	Arabidopsis	http://cressexpress.org/	Srinivasasainagendra et al., 2008
	GeneMANIA	Interactive network displaying various functional associations	Arabidopsis	http://www.genemania.org/	Warde-Farley et al., 2010
	AraNet	Probabilistic functional gene network of Arabidopsis	Arabidopsis	http://www.functionalnet.org/aranet/search.html	Lee et al., 2010
	CORNET	Co-expression analysis on predefined or user defined experiments	Arabidopsis	https://bioinformatics.psb.ugent.be/cornet/	De Bodt et al., 2010
	PLANEX	Plant gene co-expression database	Several species	http://planex.plantbioinformatics.org/	Yim et al., 2012
	Oryza Express	Gene expression database for Rice	Rice	http://bioinf.mind.meiji.ac.jp/OryzaExpress/	Hamada et al., 2011
	RiceFriend	Gene expression database for Rice	Rice	http://ricefrend.dna.affrc.go.jp/	Sato et al., 2013
	**Network Visualization Tools**				
	Cytoscape	Visualization and analysis of co-expression networks		http://cytoscape.org/	Shannon et al., 2003
	GraphViz	Visualization and analysis of co-expression networks		http://www.graphviz.org/	Gansner and North, 2000
Gene prioritization	**Gene Ontology and Enrichment Analysis**				
	Blast2GO	Identify and visualize enriched GO terms in ranked lists of genes		https://www.blast2go.com/	Conesa et al., 2005
	biNGO			http://apps.cytoscape.org/apps/bingo	Maere et al., 2005
	**Biochemical Pathways**				
	KEGG (pathways)	Collection of manually drawn pathways	Several species	http://www.genome.jp/kegg/	Kanehisa and Goto, 2000
	BioCyc	Pathway and genome database	Several species	http://biocyc.org/	Caspi et al., 2014
	Mapman	Display large data sets on diagram of metabolic maps	Several species	http://mapman.gabipd.org/	Thimm et al., 2004
	**Transcription Factors Identification**				
	plantTFDB	Plant transcription factor database	Several species	http://planttfdb.cbi.pku.edu.cn/	Jin et al., 2014
	**CIS-Regulatory Elements Enrichment**				
	PLACE	Database of motifs found in cis-acting regulatory elements	Arabidopsis	https://sogo.dna.affrc.go.jp/cgi-bin/sogo.cgi?lang=en&pj=640&action=page&page=newplace	Higo et al., 1999
	AGRIS and AtregNet	Information resource of Arabidopsis promoter sequences, Transcription factor and targets	Arabidopsis	http://arabidopsis.med.ohio-state.edu/	Palaniswamy et al., 2006
	**Text Mining**				
	PubTator	Web-based tool for accelerating manual literature curation		http://www.ncbi.nlm.nih.gov/CBBresearch/Lu/Demo/PubTator/index.cgi?user=User171748688	Wei et al., 2012
	EVEX	Large scale text mining resource		http://www.evexdb.org	Hakala et al., 2015
	**Phenotypic Information**				
	TAIR	The Arabidopsis Information Resource for mutant phenotype information	Arabidopsis	http://www.arabidopsis.org/	Lamesch et al., 2012
Comparative co-expression network analysis	ComplEX	Explore and compare sub-networks of three species	Arabidopsis, poplar and rice	http://complex.plantgenie.org/	Netotea et al., 2014
	CoExpNetViz	Comparative co-expression analysis for bait genes	Several species	http://bioinformatics.psb.ugent.be/webtools/coexpr/index.php	Tzfadia et al., 2015
	PLAZA	Database to explore gene families and genomic homology	Several species	http://bioinformatics.psb.ugent.be/plaza/	Proost et al., 2015

Co-expression networks allow the simultaneous investigation of multiple gene co-expression patterns across a wide range of conditions. As a result, publicly available transcriptome data sets represent valuable resources for such analysis. It has been reported that nearly one in four studies uses public data to address a biological problem without generating new raw data (Rung and Brazma, 2013). The reuse of such data strengthens the need for reliable expression studies. A correct experimental design, the proper execution of the wet lab experiments and thorough annotation of the data are essential prerequisites for successful subsequent reuse (Brazma, 2003).

Several gene co-expression databases are available to help researchers in their investigations (reviewed in Brady and Provart, 2009; Usadel et al., 2009; Table 1). These databases provide user-friendly interfaces to facilitate access to the data and most of them also offer integrated data processing tools. ATTED-II (Obayashi et al., 2007, 2014) allows condition specific searches for co-expressed genes in several plant species. For Arabidopsis, CressExpress (Srinivasasainagendra et al., 2008) in addition allows selection of data sets based on a quality score to filter out “bad” microarrays. GeneMANIA (Warde-Farley et al., 2010) uses a large set of functional data of various types (predicted interactions, correlations, physical interactions and shared protein domains) to display all predicted interactions for a query gene list in an interactive network. The probabilistic functional gene network AraNet (Lee et al., 2015b) provides a measure to assess the connectivity of the query genes used in regard to the generated network. Additionally, AraNet integrates enrichment analysis tools for network components for gene ontology terms and biochemical pathways (Mapman, BioCyc and KEGG) (see Section Gene Prioritization). A popular platform for network inference is Cytoscape (Shannon et al., 2003). This open source program with its many plugins and apps allows the integration, visualization and analyses of network data (Saito et al., 2012).

## Data selection for co-expression network analysis

Publicly available gene expression databases can be queried using two main approaches. These approaches are reported in the literature as “non-targeted” (or “global”) and “targeted” (or “guided-gene”) approaches (Aoki et al., 2007). The use of one or the other approach is largely determined by the biological question addressed and the available knowledge.

The non-targeted approach provides a global overview of co-expression patterns of multiple genes across many conditions. This approach is also termed knowledge-independent or condition-independent, as no *a priori* information is used to construct the network. As an example, Mao et al. (2009) built an Arabidopsis gene co-expression network using gene expression data from 1094 non-redundant Affymetrix ATH1 arrays from the AtGenExpress consortium. This data set represented nine categories of experimental conditions, such as environmental stresses, hormonal treatments and developmental stages. The resulting network consisted of 6206 nodes and 512,936 edges. These “global” networks are generally used to describe the overall set of connections predicted to occur between gene pairs. Separated modules of functionally related genes can be identified and enable further gene prioritization (see Section Gene Prioritization).

In these global networks, also designated as condition-independent, weak interactions or interactions only occurring under specific conditions are easily missed. This can be circumvented by specifically selecting data from experiments that are relevant to the biological question addressed (Saito et al., 2008; Usadel et al., 2009). The resulting condition-dependent networks provide insights on specific biological processes (Atias et al., 2009). Illustratively, by selecting 138 samples from publicly available gene expression data sets exclusively from mature imbibed Arabidopsis seeds, Bassel et al. (2011) established a seed specific network. This SeedNet enabled the identification of modules associated with seed traits such as germination and dormancy. Childs et al. (2011) reported the improved predictive power for gene functional annotation of such condition-dependent networks. One of the limits of this approach is that the elucidation of system wide properties, such as intersecting biological pathways and genes exhibiting pleiotropic effects, might be overlooked.

An alternative approach allows to mimic condition-dependent data set selection, while using the full potential of gene expression data sets. This approach consists of pre-clustering the samples prior to network construction. In this case, a clustering algorithm is directly applied to the normalized expression matrix (genes × conditions) to partition the input samples into a defined number of groups based on their overall expression similarity. Co-expression networks are then built from each of the clusters obtained. Using this technique, Feltus et al. (2013) have shown that such an unsupervised pre-clustering approach improved capturing of co-expressed genes and the representation of unique biological terms in the derived network modules.

When experimental data have elucidated key components of specific pathways, a guide-gene approach can help to identify novel members of the same pathway in a more targeted manner (Itkin et al., 2013). These known genes, also called bait or seed genes, are used as input genes to build a seeded co-expression network. For example, Yang et al. (2011) used this approach to identify new candidate genes involved in cell-wall biosynthesis. They first established a list of 121 genes known to be involved in cell-wall biosynthesis and by querying available data sets with these seed genes, the initial list was extended to 694 potential candidate genes.

Strategies combining guide-gene queries and condition-dependent approaches may empower the predictive power of co-expression networks. For instance, Li et al. (2009) implemented a pipeline based on QUBIC, a QUalitative BIClustering algorithm, to select the conditions under which seed genes of the plant cell-wall biosynthesis pathway in Arabidopsis were found to be co-expressed among a total set of 351 conditions. These conditions were then used to generate networks of co-expressed gene modules.

## Gene prioritization

Once a co-expression network is obtained, biological relevant information can be mined by gene prioritization. This process consists of integrating diverse data sources to allow the ranking of the nodes in the network and to identify groups of functionally related genes, down to important putative regulatory genes. A panel of databases and tools are available to facilitate the integration of gene information in the network (Table 1).

In nature, a variety of biological networks have displayed evidence of scale-free behavior (Barabási and Oltvai, 2004; Albert, 2005; Atias et al., 2009). Such networks are characterized by a distribution of nodes following a power law distribution. Graphically, this type of network displays a relatively large number of low-connected nodes and a few nodes with a high connectivity, the so called “hubs.” Even though, the assumption of a power law distribution is stated in numerous studies, statistical analyses have also refuted this approach (Khanin and Wit, 2006; Lima-Mendez and Van Helden, 2009).

The network topology encodes preliminary evidences for the understanding of the underpinning biological organization and reveals biological relevant information on the functional importance of individual nodes (Atias et al., 2009). Parameters derived from network local properties such as clustering coefficient, node degree (number of connected nodes), betweenness and centrality are commonly used for node ranking (Pavlopoulos et al., 2011). Nodes with a higher rank, i.e., with a high degree of connection and a high clustering coefficient, are identified as major hubs and are also likely associated to essential genes in the network (Provero, 2002; Carlson et al., 2006). The phenomenon, describing the link between connectivity and essentiality is termed the “lethality-centrality rule” (Jeong et al., 2001). Several studies have associated the non-trivial topological features of scale free networks to an essential buffering system for biological networks robustness and environmental responses (Levy and Siegal, 2008; Fu et al., 2009; Lachowiec et al., 2015).

Groups of highly connected genes in a network tend to form modules. Extracting modules from the network is thus a commonly used approach to generate manageable graph subunits for further study (Aoki et al., 2007; Mao et al., 2009). For this purpose, several clustering algorithms are available. These algorithms can be categorized into hierarchical and non-hierarchical algorithms. Hierarchical clustering algorithms identify clusters by iteratively assigning nodes to clusters. In a first step, weights are assigned to the network vertices, using for instance the calculated correlation coefficient. Clusters are then built from high weight vertices and progressively expanded by including neighboring vertices. The number of final clusters varies, for instance depending on a chosen threshold. A variety of hierarchical clustering methods are available including Weighted Gene Correlation Network Analysis (WGCNA) (Langfelder and Horvath, 2008), Markov Cluster Algorithm (MCL) (Enright et al., 2002; Mao et al., 2009), Normalization Engine for Matching Organizations (NeMo) (Rivera et al., 2010) and Improved Principal Component Analysis (IPCA) (Li M. et al., 2008; Fukushima et al., 2012). Mutwil et al. (2010) suggested a novel Heuristic Cluster Chiseling Algorithm (HCCA). For each node in the network, this algorithm generates node vicinity networks by collecting all nodes within *n* steps away from the seed node. Non-hierarchical approaches, such as K-mean clustering (Stuart et al., 2003), identify a certain number of modules given the input cluster criteria instead.

The performance of the different clustering algorithms can be assessed by evaluating the functional coherence of the predicted modules and inform, in return, the user on the best clustering algorithm to use (Lysenko et al., 2011). MORPH, an algorithm developed by Tzfadia et al. (2012), combines a guide-gene approach with data set selection and clustering to enable finding the best combination of gene expression data and network clustering to optimally associate candidate genes with a given target pathway.

Modules are often used as the starting point for more detailed studies as they considerably reduce the global network complexity. A panel of tools can be employed to further mine these modules (Table 1). These tools enable the functional annotation of nodes and modules and to unravel the nature of the gene-gene relationships.

Enrichment analysis for the genes within a module is the most widely used technique to associate modules with particular functions. Under the “guilt-by-association” rule, these functional modules provide a powerful framework for the identification of new genes relevant to biological processes and their functional annotation in the absence of strong *a priori* knowledge. These enrichment analyses mostly rely on annotation databases (Table 1). The most popular ones are the gene ontology (GO) database (Ashburner et al., 2000) and manually curated databases for metabolite pathways such as the Kyoto Encyclopedia for Genes and Genomes (KEGG) (Kanehisa and Goto, 2000), Mapman (Thimm et al., 2004), or BioCyc (Caspi et al., 2014).

Phenotypic data can also be used with the *a priori* expectation that clustered genes collaborate to control the same phenotypic trait. For example, Mutwil et al. (2010) successfully associated an individual cluster with a specific biological function using phenotypic data and tissue-dependent expression profiles for each gene in the cluster. Similarly, Ficklin et al. (2010) used phenotypic information of rice mutant lines to identify clusters of genes enriched for mutant phenotypic terms such as “sterile” or “dwarf.” In another study, Lee et al. (2010) showed that genes whose disruption is associated with embryonic lethality and pigmentation were significantly more interlinked in the AraNet network than expected by chance, corroborating the aforementioned centrality-essentiality theory.

Other available data can help to unravel the nature of the links connecting genes in the network. Co-expression networks are undirected networks as the edges between two genes do not indicate the direction of the interaction. Additionally, the co-expression link between two connected genes might also indicate an indirect interaction. To further unravel the gene regulatory dynamics in such modules, known gene-gene interactions can be displayed on the network and help to identify gene regulatory relationships (Ulitsky and Shamir, 2009).

One of the common approaches to identify regulatory relationships is to focus on known transcription factors and their known targets in the network. As transcription factors regulate the expression of many genes in the genome, one might also expect to find them as highly connected nodes in the network or connected to hub genes. The range of interactions of a transcription factor is defined by its binding capacity to specific cis-regulatory elements (motifs) identified in the promoter region of its target genes. Consequently, the search for such motifs in the nodes located in the vicinity of identified transcription factors can be a complementary source to functionally annotate genes and infer potential gene regulatory relationships (Vandepoele et al., 2009).

In their approach, Ma et al. (2013) used a bottom-up approach by first creating sub networks of genes based on motif enrichment for specific cis-regulatory elements and then identifying co-expression modules in those sub-networks.

Gene interaction information can also be retrieved from other data sources. The development and application of genome-wide methods for detecting protein-protein interactions, such as yeast two-hybrid (Brückner et al., 2009) or affinity purification methods coupled to mass spectrometry (Morris et al., 2014) have increased available interactome data. The InterProScan (Quevillon et al., 2005) or STRING (Szklarczyk et al., 2014) databases can be investigated to retrieve known physical interactions, both structurally resolved and experimentally validated. Knowledge on genetic interactions enables further inferring of functional relationships between genes and pathways. Besides data storage in databases, information on gene function and interactions can also be found embedded in textual data (Hakala et al., 2015). Text mining methods applied to literature resources, such as PubMed articles, help to extract additional information using manual curation efforts (Szakonyi et al., 2015) or semi-automated tools such as PubTator (Wei et al., 2012).

Previously mentioned data mining approaches essentially rely on available knowledge. Ample knowledge is available for Arabidopsis, but for other less well-studied plant species, the lack of knowledge regarding gene annotation and interactions severely limits network analysis using gene prioritization. Comparing networks from different species can provide an additional source of knowledge for gene functional annotation and gene connectivity using gene orthologs information and network alignment (see Section Comparative Co-Expression Network Analysis). As an example, Lee et al. (2015a) used conserved functional gene associations from networks inferred for Arabidopsis, worm, human and yeast as an additional source of data for the RiceNet, which was initially limited to rice-specific data sets.

The availability of these complementary data has opened the way to integrated approaches for function prediction studies. Multiple independent lines of evidence provide confidence for network functional gene associations. Kourmpetis et al. (2011) employed the Bayesian Markov Random Fields (BMRF) model to integrate protein sequence information, gene expression and protein-protein interaction data in their function prediction approach in Arabidopsis. They demonstrated that the model for network integration had the best performance when all of these data sources were used. One of the best examples of data integration is provided by GeneMANIA. This prediction server relies on a Gaussian Markov Random Fields-based method for protein function prediction combining multiple networks (Warde-Farley et al., 2010).

Together with computational methods, these tools, mobilizing and integrating prior knowledge and network features, have contributed to the establishment of diverse strategies to prioritize candidate genes for further experimentation (Table 2).

**Table 2:** **Examples of strategies used for co-expression network analysis in regard to the respective biological question addressed**.

**Review Sections**	**Biological question**	**Species**	**Strategy**	**References**
Data availability for co-expression network analysis	Identify functional modules associated to germination and dormancy	Arabidopsis	Use of a condition dependant approach	Bassel et al., 2011
	Build a comprehensive and functional co-expression network	Arabidopsis, rice	Integration of multiple sources of data in the network construction to support functional gene linkage	Lee et al., 2010, 2011
	Gene functional annotation	Rice	Comparison of condition dependant and condition independent network based approach.	Childs et al., 2011
	Maximize the capture of gene co-expression relationship	Arabidopsis	Pre-clustering of input expression samples to approximate condition dependant approach	Feltus et al., 2013
Gene prioritization	Explore the modular biological organization	Arabidopsis	Arabidopsis gene co-expression network based on 1000 microarrays. Modules were extracted using the Markov Clustering Algorithm (MCL)	Mao et al., 2009
	Infer gene regulatory relationships in gene co-expression modules	Arabidopsis	Identify gene expression modules driven by known *cis*-regulatory motifs	Ma et al., 2013
	Gene functional annotation	Arabidopsis	Module enrichment for known *cis*-regulatory elements	Vandepoele et al., 2009
	Identify co-expression modules	Arabidopsis	Development of an Heuristic clustering algorithm	Mutwil et al., 2010
eQTL based co-expression networks	Identify causal genes responsible for glucosinolate variation	Arabidopsis	Use co-expression network as non-genetic (independent) filter to prioritize GWA mapping candidates	Chan et al., 2011
	Identify candidates for shade avoidance	Arabidopsis	Prioritize genes underlying phenotypic QTL using co-expression network analysis, eQTL information and functional classification	Jimenez-Gomez et al., 2010
	Examine natural variation in circadian clock function	Arabidopsis	eQTL mapping using *a priori* defined phase groups and comparison with metabolomics QTLs	Kerwin et al., 2011
	Examine transcriptional network response to biotic interactions	Arabidopsis	Perform a network eQTL analysis from *a priori* defined gene expression networks	Kliebenstein et al., 2006
	Identify novel abiotic stress genes	Arabidopsis	Network guided genetic screen: gene ranking combined to co-expression network analysis	Ransbotyn et al., 2014
Temporal resolution for co-expression network	Resolve the chronological regulatory mechanisms involved in the response to pathogen infection	Arabidopsis	Temporal clustering by combining extensive time series data and co-expression network analysis	Windram et al., 2012
	Identify key genes regulating the acquisition of longevity during seed maturation	Medicago Arabidopsis	Developmental time course data and cross species comparison for co-expression network analysis	Righetti et al., 2015
Spatial resolution for dynamic co-expression network	Identify cell-specific molecular mechanisms	Maize	Combine Laser-capture microscopy with RNA-seq	Zhan et al., 2015
Comparative co-expression network analysis	Knowledge transfer between species	Maize rice	Global co-expression network alignment using both gene homology and network topology	Ficklin and Feltus, 2011
	Identify conserved modules across species	Several species	Co-expressed node vicinity networks (NVNS) compared across species.	Mutwil et al., 2011

## Co-expression network applications

### eQTL based co-expression networks

Advances in “genetical genomics” have greatly benefited the elucidation of the genetic loci controlling transcription and the inference of regulatory mechanisms underlying complex phenotypic traits. The concept of “genetical genomics” was first introduced by Jansen and Nap in 2001 (Jansen and Nap, 2001), marking a new turn in genetic studies. The basic idea of this approach is to join classical genetic linkage analysis (Quantitative trait Loci (QTL) analysis) with gene expression studies (Keurentjes et al., 2007). The variation in gene expression is regarded as a quantitative trait for which the genetic basis (expression QTL, eQTLs) is investigated in mapping populations, such as recombinant inbred line (RIL) populations. In plants, “genetical genomics” has proven to be a successful strategy to dissect complex traits in a number of studies (for reviews see Joosen et al., 2009; Kliebenstein, 2009; Ligterink et al., 2012).

Detected eQTLs for a specific gene can be classified into “local” or “distant” eQTLs depending on whether they co-localize with the physical position of the studied gene or are located elsewhere in the genome, respectively (Rockman and Kruglyak, 2006). eQTLs can also be classified as *cis*- or *trans*-acting based on the location of the associated causal polymorphism in the gene under study or elsewhere in the genome, respectively. Consequently, distant eQTLs are always *trans*-acting, while local eQTLs can be *cis*-acting, if the associated causal polymorphism resides in the gene under study, or *trans*-eQTLs when they are caused by a closely linked allelic variation in a *trans*-acting factor. Allele specific expression analysis can specifically determine whether a local eQTL is *trans* or *cis*-acting (for review see Kliebenstein, 2009).

A common feature of global eQTL studies is the identification of *trans*-eQTL hotspots (Keurentjes et al., 2007; West et al., 2007). These eQTL hotspots correspond to a high number of co-locating *trans*-eQTLs in one region of the genome, indicating a hotspot for transcriptional regulation (Kliebenstein, 2009). Due to their analogy to high degree nodes in a network, *cis*-eQTLs located in these hotspots are sought as candidate master regulators affecting the expression of genes with a *trans*-eQTL in that same region (West et al., 2007). A regulatory relationship can be inferred by correlating gene expression profiles between the *cis*-eQTL candidate regulators and their potential downstream *trans* regulated genes. An iterative group analysis can be used to detect significant associations (Breitling et al., 2004; Keurentjes et al., 2007; Wang et al., 2014). Keurentjes et al. (2007) established a regulatory network for genes involved in the transition of flowering based on eQTL data. The GIGANTEA (GI) protein, known to be involved in the circadian clock controlled flowering time pathway, was identified as a regulator. Phenotypic QTLs associated with flowering and the circadian clock were also identified at the genetic locus of *GI*. Similarly, Wang et al. (2014) identified eight regulatory groups and their target genes for heading time in rice RILs. One regulatory group centered on *Ghd7*, an important regulator in heading time and yield potential in rice, was identified with a *cis*-eQTL connected to nine genes with *trans*-eQTLs. The network was validated by inspecting the transcript abundance of downstream-regulated targets and supported by co-localizing phenotypic QTLs for yield and heading time. These studies illustrate the usefulness of eQTL based co-expression analysis to guide the identification of candidate genes controlling quantitative traits. Other studies combined eQTL with co-expression analysis to identify regulator candidates underlying eQTLs (Terpstra et al., 2010; Flassig et al., 2013).

Interestingly, eQTL studies have also reported noteworthy properties of eQTLs in regard to their regulatory and evolutionary significance. *cis*-eQTLs were found to be highly inheritable with a larger genetic effect when compared to *trans*-eQTLs (Petretto et al., 2006; West et al., 2007; Kloosterman et al., 2012). *cis*-eQTLs were also found to be more consistent across different genetic backgrounds (Cubillos et al., 2012) and more robust to environmental perturbations (Cubillos et al., 2014), while genes with *trans*-eQTLs were more frequently reported as tissue or organ specific (Drost et al., 2010; Kloosterman et al., 2012).

QTLs tend to cover large regions of the genome, typically spanning hundreds of genes, and finding the actual gene that causes the observed trait variation is a formidable task. The capacity of gene co-expression networks to handle genome-wide data and filter out genes based on their correlation coefficients offers an attractive approach to prioritize genes. This strategy was successfully applied in the identification of *EARLY FLOWERING 3* (*ELF3*), and its implication in shade avoidance response (Jimenez-Gomez et al., 2010). In this study, a network was built for each of the 363 candidate genes underlying the main phenotypic QTL for shade avoidance, connecting each candidate gene to co-expressed genes across 1.388 (selected) experiments. The eQTLs available for the investigated RIL population allowed pruning of the networks to keep only the co-expressed genes with a *cis*-eQTL, which is indicative of a regulatory relationship (Hansen et al., 2008). In a similar approach, Chan et al. (2011) used co-expression analysis to prioritize candidate genes resulting from a genome wide association study (GWAS). Alternatively, co-expression networks can be used prior to eQTL analysis (Kliebenstein et al., 2006; Kerwin et al., 2011). Kliebenstein et al. (2006) implemented an *a priori* network eQTL approach by calculating the mean expression value of the genes within each pre-determined network and using this as a quantitative trait in a subsequent QTL analysis.

**Figure 2:**
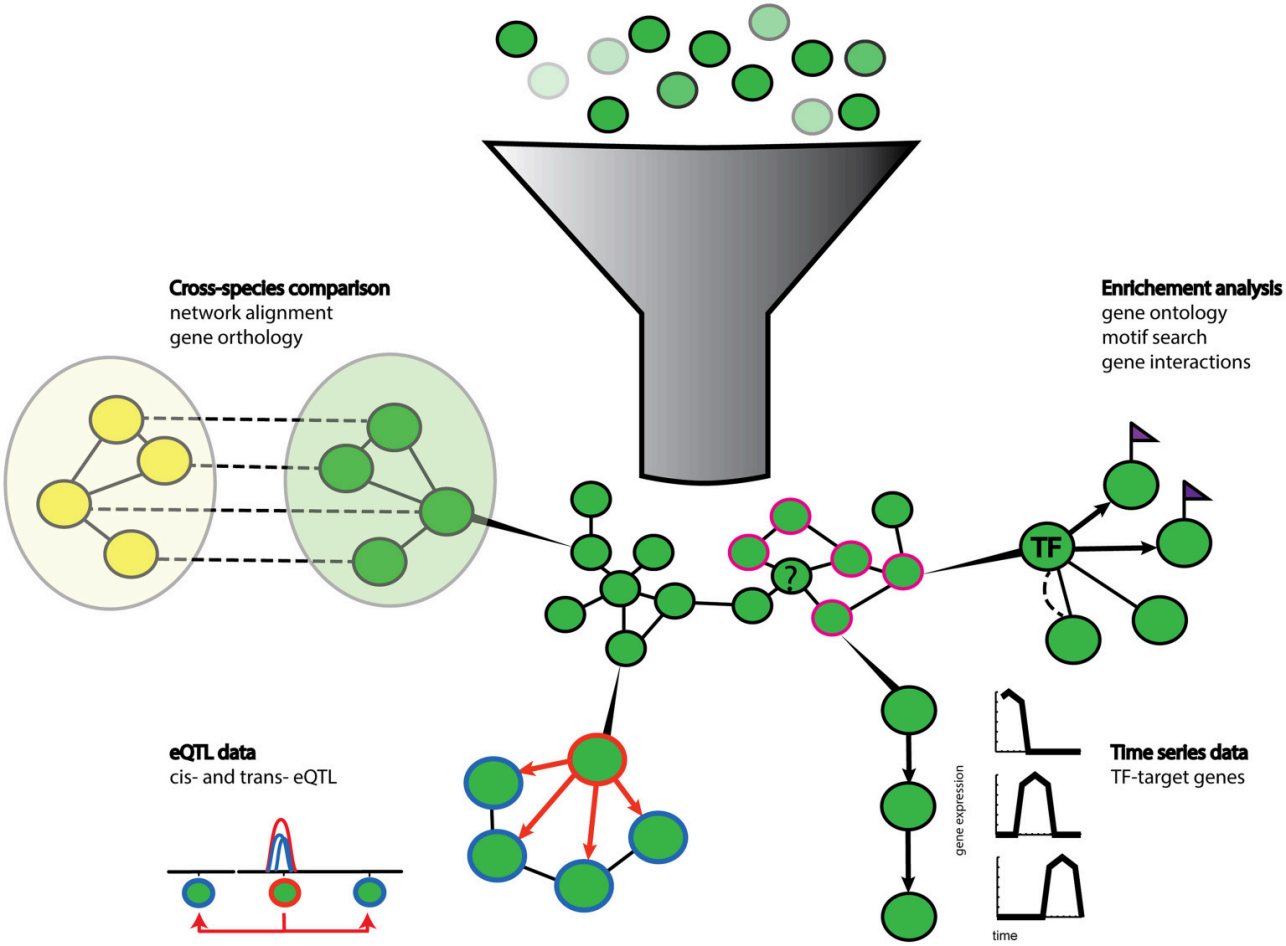
**Schematic representation of gene prioritization strategies**. Gene sets of different expression values (shades of green) are used for co-expression network inference. Genes with co-expression values above a user defined threshold (dark green nodes) form nodes and edges in the network. Various additional data can then be used to enrich and extract biological relevant information from the network. Enrichment analysis tools such as gene ontology terms (pink contour nodes) can be used to functionally annotate unknown genes (question marked node) clustered in the vicinity. Prior knowledge can also help to highlight known gene-gene interactions (dotted line) and cis-regulatory motif (purple flags) can suggest local regulatory interactions (arrows) between transcription factors (TF node) and their target genes (flagged nodes). Gene regulatory relationships can also be extracted from time series data. Algorithms can extract causal regulatory relationships from shifted gene expression patterns in time series data. Co-localization of trans- and cis-eQTLs (hotspots) can also infer regulatory relationships between genes with a cis-eQTL (orange contour node) and genes with trans-eQTLs (blue contour node). Additional information can be gained from comparisons with networks of other species (yellow nodes) by orthology and network alignment (dotted lines).

One main advantage of eQTL analysis is that regulatory insights can be gained without prior knowledge. Information on the nature of the inferred interaction in such an approach, combined with co-expression network analysis, can substantially accelerate understanding of molecular regulatory interactions (Figure 2). However, the link between phenotype and transcript variation is not always straightforward as changes are also likely to occur at the protein or metabolite levels. The additional integration of other omics data available as QTLs for protein (pQTL) or metabolite (mQTL) variation (Wentzell et al., 2007; Kerwin et al., 2011) can bridge the gap between genotype and phenotype, providing an in-depth understanding of causal mechanisms. As an example, Kerwin et al. (2011) identified overlapping eQTLs and mQTLs for circadian time and glucosinolate variation in Arabidopsis. Specifically, AOP2, a 2-oxoglutarate-dependent dioxygenase, was identified as a potential regulator. Altered AOP2 function resulted in changes in expression of clock output genes, suggesting a causal relationship between changes in clock function and metabolite content.

### High-resolution co-expression networks

Co-expression networks offer a conceptual framework to study gene interactions. However, their static representation does not capture all possible gene relationships as these do not operate simultaneously due to spatial and temporal variation in gene expression.

#### Temporal resolution for dynamic co-expression networks

In response to developmental or environmental stimuli, plants undergo global transcriptional reprogramming. Monitoring transcriptional changes over time can provide more insight into the cascade of biological processes involved in the signal perception, transduction and final response.

Using time series data sets throughout seed development, Le et al. (2010) identified seed specific transcription factors active in different compartments and tissues of the seed at unique moments of seed development, suggesting a chronology of specific regulatory programs triggering seed development.

Time series experiments are often used to examine the dynamics of gene expression. Wei et al. (2013) used six time points during growth of poplar roots in low nitrogen conditions. GO categories associated with signal transduction were identified for differentially expressed gene sets in the early time points of the response (6 and 24 h), while categories associated with organ morphogenesis were prevalent throughout the later time points (48 and 96 h). By reducing the time scale to minutes, Krouk et al. (2010) observed that within 3 min following nitrate addition in Arabidopsis, functional categories such as ribosomal proteins were over-represented, suggesting the rapid activation of key elements of the translation machinery to synthesize proteins required for nitrogen acquisition. Combining time series and co-expression network analysis can unveil gene interactions associated with the dynamics of transcriptional programs. Global expression patterns can be obtained from the expression similarity calculated across samples collected at different time points. This approach is well suited to find modules of simultaneous expressed genes and gene interactions but is not well suited for time lagged regulations since all genes influencing the expression of downstream target genes are not necessarily captured within a same time point (experiment). This results in complex relationships between co-regulated genes, including co-expression, time shifted and inverted relationships (Zhang et al., 2005): an activated transcription factor gene first has to be transcribed and the resulting mRNA translated before it in turn can activate its downstream targets. The delay further depends on the dynamics of the regulation, and for instance the presence of network motifs like feed forward or negative feedback loops (Alon, 2007).

Windram et al. (2012) dissected the infection response of Arabidopsis to *Botrytis cinerea* using 48 time points with 2-h intervals. To capture the chronological establishment of the associated transcriptional events and to predict their regulation, the differentially expressed genes were first clustered based on the similarity of gene expression patterns over time or based on the timing of differential expression of each gene. Regulatory predictions were made using a discrete-time causal structure identification algorithm. The expression means of the clusters and *Botrytis cinerea* growth information were used to build a regulatory network. In this network, a NAC transcription factor identified in one cluster connected to two downstream clusters enriched for the NAC binding motif in their promoter sequence, suggesting a regulatory relationship.

This example shows that causality information of time series on a fine temporal scale can provide valuable information on the directionality of gene interactions. Several algorithms have been proposed to perform time delayed correlation analysis in time series data (De Smet and Marchal, 2010). For instance, Lavenus et al. (2015) proposed a time delay correlation algorithm (TDCor) that includes minimal prior knowledge on the nature of the genes, with transcription factors categorized as repressor, activator, regulator or non-regulator, to build a network of plausible interactions from time series data. Krouk et al. (2010) used a noise reduction state-space modeling algorithm to build a dynamic linear model defining the rate of change in expression between time points *t* and *t* + 1. This model was then used to predict the influence of transcription factors on the genes they regulated (influential rate). The authors reasoned that the observed low influential rate of the transcription factors could be due to the functional redundancy that is often observed in biological networks and is consistent with a proposed global buffering system counteracting stresses and evolutionary forces (Fu et al., 2009). Polanski et al. (2014) suggested a module identification procedure based on the Wigwams algorithm capable of mining multiple time series for condition dependent co-expression across a subset of time series. Using such an approach, the reconstruction of co-expression networks can be directed to time specific modules of co-regulated genes.

Together, these studies suggest that new regulatory insights can be gained from integration of co-expression networks with data from time series, for the identification of “subtle” gene clusters, showing condition dependent regulation. Time series are valuable for further disentangling of real co-regulatory gene relationships from co-expression links. For application in more studies, new challenges have to be addressed such as the judicious selection of time points (Vashishtha et al., 2015), the development of performant inference algorithms, the reliable detection of direct and indirect gene interactions and most importantly the connection with their real biological meaning (reviewed by Bar-Joseph et al., 2012). We believe that this approach will offer new venues for deeper insights into the fine-tuned regulation and predictive analysis of gene expression behavior in future studies.

#### Spatial resolution for dynamic co-expression networks

Plants are multicellular organisms whose vegetative and reproductive organs are composed of complex tissues and cell types. Cell differentiation is a fundamental process required to acquire cell identity and consequently ensure the correct execution of essential structural and biological functions. Genome-wide transcriptome and gene network analyses have mostly been conducted on whole plant organs, severely limiting the identification of more specific regulatory interactions occurring at the tissue or single cell level. The development of new highly selective methods has enabled the collection of expression profiles at unprecedented resolution (Nelson et al., 2008; Tang et al., 2011; Belmonte et al., 2013) offering new insights into the various biological levels of transcription regulation. As an example, laser capture microdissection (LCM) enables isolation of specific tissues at cell level while fluorescent activated cell sorting (FACS) allows separation of specific cell types expressing green fluorescent protein (GFP) under control of cell specific promoters.

These techniques were used to get insight into single cell transcriptomic data for well-studied and specialized organs such as roots or pollen (Aya et al., 2011; Becker et al., 2014; Slane et al., 2014; Efroni et al., 2015).

A fluorescent cell sorting technique was used to obtain a high-resolution map of spatiotemporal expression profiles of Arabidopsis roots (Brady et al., 2007). In this study, transcriptome analysis of root transverse sections revealed 51 dominant root radial expression patterns among which 17 showed enrichment in a single cell type, whereas 34 expression patterns were found across 2–5 cell types (Brady et al., 2007). In the same study, the longitudinal root section expression profiling to analyse different developmental stages in root cell-type formation, enabled the identification of specific expression patterns. Transcriptional changes may also occur in response to environmental shifts. Interestingly, a close link was observed between development and stress responses at the cell-type specific level in the Arabidopsis root showing developmental plasticity (Gifford et al., 2008) while adding a layer of complexity, i.e., environment specific effects, to an already intricate system. Together, these results highlight the spatiotemporal transcriptional complexity down to the cellular level and suggest cell-specific transcriptional programs.

Integrating tissue- or cell-type specific high-resolution datasets by co-expression network analysis is a promising approach for the regulatory dissection of specific biological functions. Illustratively, Zhan et al. (2015) combined LCM and RNA-seq to isolate and profile filial and maternal cell types of maize kernels at 8 days after pollination. From the resulting gene co-expression network, 18 endosperm-associated co-expression modules were identified among which 10 were found to be highly compartment- or cell-type-specific. The comparison of these spatial co-expression modules with temporally upregulated gene data sets showed that genes within co-expression modules are regulated both in time and space. Collectively, these results support the effectiveness of co-expression networks analysis to uncover the temporal and spatial organization of specific differentiation processes.

On-going developments to further improve single-cell RNA-seq analysis (Buettner et al., 2015) should strongly benefit the establishment and interpretation of specialized co-expression networks in the coming years. Furthermore, the advancement of computational tools able to manage the increasing amount of data as well as the development of robust and efficient algorithms to analyse large-scale data will be needed to tackle the increasing complexity added to gene regulatory networks.

### Comparative co-expression network analysis

“*Nothing in biology makes sense except in the light of evolution”* (Dobzhansky, 1973).

Classic research in evolutionary developmental biology (“evo-devo”) has focused on comparative analysis with the help of mutant analysis, heterologous mutant complementation, comparative gene expression studies and phylogenetic analysis. These analyses mostly rely on gene and protein sequence information; however the increasing number of gene expression data in many different species is opening up new perspectives. Cross-species comparison of co-expression networks is a promising approach to understand the interplay between regulatory function and evolution (Movahedi et al., 2012; Hansen et al., 2014).

There are several advantages of cross-species network comparisons. Networks of well-studied plants such as Arabidopsis can enrich sparse networks, such as for crops, reducing the need of extensive functional genomic and phenomic resources. Cross-species comparison can accelerate the functional annotation of genes and the discovery of gene-gene interactions, consequently hastening the gene prioritization process for targeted mutational studies.

There is evidence that networks are shaped by major evolutionary features, such as by neo- or sub-functionalization following whole genome duplications (Conant and Wolfe, 2006; De Smet and Van De Peer, 2012). These adaptive processes may result in an evolutionary functional gene network partitioning associated with a rewiring in the gene regulatory circuitry (Conant and Wolfe, 2006). In this context, co-expression network comparison can be used to identify functionally conserved network patterns and to study their evolution.

Different methods have been proposed to compare co-expression networks. Leal et al. (2014) compared gene co-expression networks obtained for several plant species in reponse to different pathogens using a multivariate analysis. Each network was characterized by eight graph variables which were then summarized in a principal component analysis. Clustered networks identified in the principal component analysis plot suggested similar pathogen specific responses across species.

An obvious method to align networks and to get better insight into the degree of network conservation is to link orthologous genes between different species. The effectiveness of such comparative analysis essentially relies on the consistency of the orthologous information as well as the quality of the underlying co-expression networks. Orthologous gene information can be obtained through various methods (Kuzniar et al., 2008). Simple approaches use best Blast hits or reciprocal hit blast (RHB) for closely related species (Yang et al., 2011). More advanced tools such as the OrthoMCL clustering algorithm (Li et al., 2003) or OrthoFinder (Emms and Kelly, 2015) enable differentiation of true orthologous from paralogous genes. Zarrineh et al. (2011) proposed a cross-species co-clustering approach (COMODO). Network comparisons can be done at the global scale or focused on specific gene modules. In a global approach, Ficklin and Feltus (2011) used an alignment algorithm, IsoRank, that incorporates both gene homology and network topology to compare networks in rice and maize. They identified aligned modules enriched for similar functional terms, suggesting their potential evolutionary conservation.

In another study, Obertello et al. (2015) used orthologous information from OrthoMCL and BlastP, to align genes between Arabidopsis and rice co-expression networks. The authors observed that integrating rice data in an Arabidopsis network did not improve the available interaction knowledge, while Arabidopsis could substantially enrich rice network interactions. This study illustrates the usability of network comparisons to promote translational discoveries. It shows that well-known networks, such as those from model plants like Arabidopsis, can enrich more sparse networks of crops, such as rice, although Lee et al. (2011) demonstrated a higher accuracy for a rice network, RiceNet, derived from data of diverse species (with 15.5% of true positive linkages) than for a rice network derived solely from orthology with AraNet, the Arabidopsis network (with 6.5% true positive linkages).

In a more targeted approach, Yang et al. (2011) investigated conserved co-expression of cell-wall associated genes between Arabidopsis and poplar. An initial list of known cell-wall related genes was used to build a co-expression network with 22 clusters. The orthologous clusters of co-expressed genes identified in poplar did not all correlate in gene expression pattern with the clusters in Arabidopsis (gene expression pattern correlated for 9 of 22 clusters). Additionally, conserved co-expression clusters referred to plant essential biological functions, such as cell-wall formation. More comprehensively, Movahedi et al. (2011) implemented an expression context conservation score (ECC) to quantitatively estimate the degree of conservation of expression similarity between orthologous genes and their co-expression partners. The overall ECC scores revealed that for 4.630 orthologs in rice-Arabidopsis gene pairs, 77% had a conserved expression context. In another study, Netotea et al. (2014) performed an extensive examination of network properties, like node degree distribution and gene centrality, to compare co-expression networks of Arabidopsis, poplar and rice. They analyzed the degree of conservation of gene co-expression links and neighborhood (connected genes) among all orthologs in the three networks and showed that genes with high centrality, typically hubs, were significantly conserved while local regulatory motifs were relatively less well conserved across species.

Additionally, they noted that sequence similarity did not always predict gene regulation conservation. Beyond simple gene sequence comparison, the integration of co-expression networks to cross-species data provides a new dimension in evolutionary studies, revealing conservation and divergence in the regulation of genes.

At the moment, several integrative platforms are available to enquire, display and compare co-expression networks. Examples of these are PLANEX (Yim et al., 2012), ComPLex (Netotea et al., 2014), CoExpNetViz (Tzfadia et al., 2015), PLAZA (Proost et al., 2015) and the “NetworkComparer” pipeline on the PlaNet platform (Mutwil et al., 2011) that integrates genomics, transcriptomics, phenomics and ontology analyses to compare seven plant species.

## Conclusion and perspectives

Co-expression networks are a powerful approach to accelerate the elucidation of molecular mechanisms underlying important biological processes. Importantly, network based strategies are largely determined by the biological question addressed and the prior knowledge available.

We anticipate that the increase in available experimental data, driven by new molecular techniques, will enrich existing databases. In addition, the shift from microarrays to next generation high-throughput sequencing technologies will provide further insights into genome scale functional networks of many species. Together with the increased sensitivity of high-resolution technologies enabling the acquisition of cell-specific transcriptome profiles, novel biological insights can be gained. The extensive accumulation of data will require further efforts for their storage, accessibility and processing. One of the common strategies for all co-expression network studies is the integration of disparate data sources for the biological interpretation of networks. As a result, the development of integrative web interfaces such as CressInt (Chen et al., 2015) are needed to facilitate the integration of available genomics data. Furthermore, the development of computational tools, such as machine learning based algorithms, although computationally intense, will support the optimal integration and exploitation of prioritization strategies (Radivojac et al., 2013). In such a scenario, the collaboration of bioinformaticians and biologists is highly desirable and will become increasingly important.

To fully describe the link between genotype and phenotype and to understand the underlying gene regulation, coordination of networks at different molecular levels (gene, protein, metabolite) is needed (Gaudinier et al., 2015). Additionally, genetically anchored gene expression profiles (eQTLs) have proven to be powerful tools to reveal causal regulatory variants. The genetical genomics approach provides a multifactorial design to study the simultaneous effect of gene perturbations. Kliebenstein (2012) demonstrated that shallow sequencing depth in transcriptomics experiments enables capturing most of their genomic information. The result of their study suggested that 10% of the transcripts would detain more than 80% of the information present in a variety of transcriptomics experiments. In another study, Li Y. et al. (2008) introduced the generalized genetical genomics design to optimally study genetic by environment interactions. These findings suggest that there is room for improvement in the design of transcript sequencing for large-scale factorial analysis in which the size of the population studied or the number of conditions to be tested can be increased in a cost-effective manner.

Co-expression networks are an attractive framework for gene interaction analysis and offer a diverse range of applications, from the gene functional annotation to the comparison of co-expression networks across species. Improved and enriched co-expression network analyses will further empower the predictive power of networks and their translational application by circumventing the need of additional extensive functional genomic and phenomic resources. This approach will further contribute to the elucidation of important biological processes and provide a valuable predictive tool for contemporary molecular breeding and crop engineering strategies.

## Author contributions

ES wrote the manuscript. HN, HH, and WL participated in the design and critical reviewing of the manuscript.

## Funding

This publication was supported by the Dutch Technology Foundation STW, which is part of the Netherlands Organization for Scientific Research (NWO), and which is partly funded by the Ministry of Economic Affairs.

### Conflict of interest statement

The authors declare that the research was conducted in the absence of any commercial or financial relationships that could be construed as a potential conflict of interest.
